# Post-vaccination antibody evaluation for nosocomial SARS-CoV-2 delta variant breakthrough infection

**DOI:** 10.1371/journal.pone.0272056

**Published:** 2022-07-25

**Authors:** Takeyuki Goto, Naoki Tani, Hideyuki Ikematsu, Kei Gondo, Ryo Oishi, Junya Minami, Kyoko Onozawa, Hiroyuki Kuwano, Koichi Akashi, Nobuyuki Shimono, Yong Chong

**Affiliations:** 1 Medicine and Biosystemic Science, Kyushu University Graduate School of Medical Sciences (The First Department of Internal Medicine), Fukuoka, Japan; 2 Ricerca Clinica Co., Fukuoka, Japan; 3 COVID-19 Team, Fukuoka City Hospital, Fukuoka, Japan; 4 Department of Infectious Diseases, Fukuoka City Hospital, Fukuoka, Japan; 5 Center for the Study of Global Infection, Kyushu University Hospital, Fukuoka, Japan; Waseda University: Waseda Daigaku, JAPAN

## Abstract

Waning humoral immunity after mRNA vaccination against severe acute respiratory syndrome coronavirus 2 (SARS-CoV-2) is a significant problem for public health. Breakthrough infection in hospitals over several months after vaccination has not been fully characterized, especially against the delta (B.1.617.2) variant. Here, we describe an outbreak in our hospital in September of 2021, mainly through serological evaluation of the breakthrough infection. This retrospective observational study was done at an emergency and acute care hospital with 204 beds and 486 staff members where most staff members (92.6%) had had their second BNT162b2 vaccination by May of 2021. The peri-infection anti-spike RBD protein IgG (anti-S IgG) titers (lowest values between 11 days before and 7 days after onset or diagnosis) of serum samples from the breakthrough-infected persons were quantified. We also logarithmically estimated the anti-S IgG titers during the exposure period in September of uninfected staff members from their samples collected in May and December 2021. Whole-genome sequencing was done on obtained samples. In this outbreak, twelve persons (ten inpatients and two staff members) were diagnosed with SARS-CoV-2 infection by Loop-Mediated Isothermal Amplification (LAMP) or RT-PCR, eight of whom had been vaccinated twice. Peri-infection anti-S IgG titers could be determined in seven of the eight breakthrough cases, with a geometric mean titer (GMT) of 1,034 AU/ml (95% confidence interval [CI], 398 to 2,686). Among 289 uninfected staff members with data from the two sampling points, the GMT of the estimated anti-S IgG titers during the exposure period in 51 staff members, who were working at the outbreak ward and potentially exposed but uninfected, and 238 other unexposed staff members were 1,458 AU/ml (95% CI, 1,196 to 1,777) and 1,628 AU/ml (95% CI, 1,500 to 1,766), respectively. All viruses from the eight samples for which whole-genome sequencing was available were identified as delta variants. Of the infected persons, one remained asymptomatic throughout the course of treatment, and eleven had an illness of mild to moderate severity, including ten who received monoclonal antibody cocktail (Casirivimab/imdevimab) therapy. Measurement and estimation of anti-spike antibody levels after SARS-CoV-2 vaccination would be helpful for evaluating the risk of breakthrough infection and for determining the necessity of booster vaccination.

## Introduction

Waning mRNA vaccine effectiveness against severe acute respiratory syndrome coronavirus 2 (SARS-CoV-2) infection has been reported worldwide and is of great concern [1–3], with decreased humoral immunity over time having been reported as an associated factor for breakthrough infections [4]. In Japan, most healthcare workers had received their second vaccination by May of 2021, and approximately four months later, they were faced with a midsummer pandemic wave caused by the delta (B.1.617.2) variant. Although nosocomial breakthrough infections of the delta variant after the second vaccination have been reported [5], their features have not been fully clarified, especially in serological analyses that include infected and uninfected persons.

In September of 2021, a SARS-CoV-2 outbreak involving breakthrough infection occurred in a hospital in Japan. The hospital has ongoing longitudinal cohort studies of its staff members which investigated the relationship between antibody titers and adverse reactions after SARS-CoV-2 vaccination, and the impact of antipyretic drug use. In this retrospective, observational study, we describe the characteristics of the breakthrough infection, chiefly through serological evaluation by measuring and estimating antibody levels.

## Methods

### Hospital information and ethics statement

This study was done at Fukuoka City Hospital, a secondary emergency and acute care hospital in Japan with 204 beds and 486 staff members, 450 (92.6%) of whom were vaccinated twice with BNT162b2 by May of 2021.

This study was approved by the ethics review board of Fukuoka City Hospital (approval number 231), which included the statement that individual consent is not required and opt-out can be acceptable because the data is anonymized. The information of this study was provided to the participants on the hospital homepage and consent was obtained.

### Participants and term definitions

SARS-CoV-2 infection was defined as a positive result on Loop-Mediated Isothermal Amplification (LAMP; Loopamp, Eikenkagaku, Tokyo, Japan) or RT-PCR assay. The severity of the disease was classified according to the National Institutes of Health (NIH) criteria [6], in which the borderline between moderate and severe is when the oxygen saturation (SpO2) is 94% on room air. Close exposures were defined as inpatients in the same room as an infected inpatient but who tested negative for SARS-CoV-2 infection. All staff members working on the same floor during the outbreak but without SARS-CoV-2 infection were defined as potential exposures. Clinical data was gleaned from the charts of all infected persons, close exposures, and the family of staff members related to this outbreak who had confirmed SARS-CoV-2 infection. The antibody data of staff members with two samples collected in May and December 2021 for our previous and ongoing cohort studies [7], and with no history of SARS-CoV-2 infection between the two sampling points were used to estimate antibody titers during the exposure period. One sample was collected after the second vaccination when the antibody levels would be expected to be at their peak and another before the third vaccination, a booster given in December of 2021.

### Serum antibody measurement and estimation

Serum samples were measured for both anti-spike RBD protein IgG (anti-S IgG) and anti-nucleocapsid protein IgG (anti-N IgG) with SARS-CoV-2 IgG Ⅱ Quant and SARS-CoV-2 IgG Reagent Kit (Abbott, Lake Forest, IL, USA), respectively, with positive cut-offs of ≧ 50 AU/ml and ≧ 1.4 Index (S/C). The peri-infection titer was defined as the lowest titer measured from the eleven days before to seven days after the onset or diagnosis. The anti-S IgG titers of staff members during the exposure period were estimated from a line graph connecting the titers at the two sampling points. This estimation is based on the assumption that the reduction ratios would be constant (i.e., the reduction of anti-S IgG is linear in a single-logarithmic graph) for a person regardless of the elapsed time, as was proposed in a previous study [4]. Data analysis was done using R (version 4.1.1, R Core Team) with the tidyverse package (version 1.3.1).

### Sequencing analysis

Specimens for viral sequencing were collected as nasopharyngeal swabs by trained physicians. After RNA extraction and DNA synthesis, target enrichment was done using Alt_nCov2019_primers ver. N3 (Itokawa. K, https://github.com/ItokawaK/Alt_nCov2019_primers/tree/master/Primers/ver_N3). The libraries were prepped with the QIAseq FX DNA Library UDI Kit (Qiagen, Hilden, Germany) for sequencing with the MiSeq Reagent Kit v2 (Illumina, San Diego, CA, USA). The obtained reads were mapped to the original SARS-CoV-2 reference genome (GISAID, hCoV-19/Wuhan/WIV04/2019/EPI_ISL_402124) using the BWA-MEM algorithm. The variant assignment was performed on Pangolin, UCSC Genome Browser (https://genome.ucsc.edu/cgi-bin/hgPhyloPlace) and Nextclade (http://clades.nextstrain.org/). The GATK variants were filtered to at least 10x depth. Data analysis was done using Python Ver 3.8.8.

## Results

**Fig 1:**
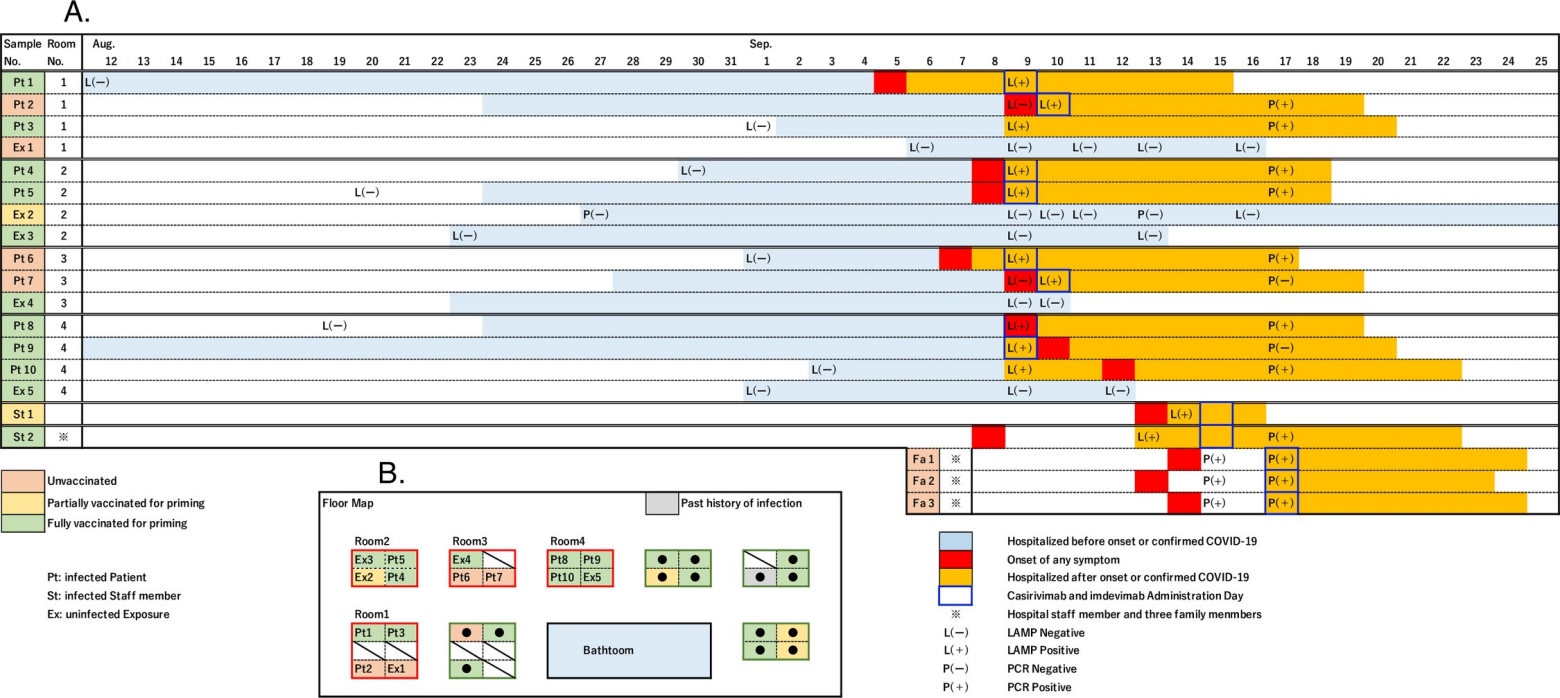
(A) Time course of the nosocomial spread of COVID-19 and the spread within one family. (B) Bed map showing the spread within a single ward. Time course and bed map are shown for each room, colored for the vaccination status. The admission period, onset date, duration of admission, LAMP and PCR results, and administration of monoclonal antibody cocktail (Casirivimab/imdevimab) therapy are shown in the progress chart.

The clinical characteristics of the infected persons and close exposures are shown in Table 1, and the time course of the nosocomial COVID-19 spread is shown in Fig 1. In the outbreak in September of 2021, 10 of 29 inpatients in the same ward and 2 of 107 staff members who worked with them were diagnosed with SARS-CoV-2 infection. The onset of the outbreak was limited to the period between September 5 and 13, 2021. Five inpatients were defined as close exposures, and 105 staff members were defined as potential exposures. All three family members living with one of the infected staff members were confirmed to be infected. Of the ten infected inpatients and two staff members, eight (67%) were 2-dose vaccinated, one (8%) had 1-dose, and three (25%) were unvaccinated. All vaccinations were conducted using the BNT162b2 vaccine. One inpatient who had been vaccinated twice did not develop any symptoms, but all other infected persons eventually developed COVID-19-related symptoms within the mild to moderate categories. Except for two inpatients with no or mild to moderate symptoms, ten received monoclonal antibody cocktail (Casirivimab/imdevimab) therapy.

**Table 1:** Clinical characteristics, vaccine history, and symptoms associtated with COVID-19 of infected persons and uninfected close exposures.

Sample	Sex	Age	BMI	Comorbidities^a^	Vaccination status	Interval between onset or exposure and last vaccination	Peri-infection^b^ or peri-exposure^c^ S-IgG titer	Symptoms	Administration day of antibody therapy from onset	Severity / Clinical Outcome
Patient 1	Male	69	27.7	Hypertension, Smoker	Twice	55 Days	No data	Fever, cough, headache, pharyngeal pain	Day 4	mild to moderate / Recovered
Patient 2	Male	56	23.4	Diabetes Hypertension	-	-	1.8 (AU/mL)	Fever, pharyngeal pain	Day 1	mild to moderate / Recovered
Patient 3	Male	54	27.7	Diabetes Hypertension	Twice	38 Days	5013 (AU/mL)	-	-	asymptom
Patient 4	Female	84	22.1	Chronic kidney disease Diabetes, Hypertension Chronic herat failure	Twice	61 Days	1027 (AU/mL)	Fever	Day 1	mild to moderate / Recovered
Patient 5	Female	78	27	Hypertension Past smoker	Twice	78 Days	399 (AU/mL)	Fever, cough, headache, pharyngeal pain, rhinorrhea	Day 1	mild to moderate / Recovered
Patient 6	Male	48	32.8	-	-	-	1.8 (AU/mL)	Fever	Day 2	mild to moderate / Recovered
Patient 7	Male	40	34.1	Smoker	-	-	2.1 (AU/mL)	Fever, pharyngeal pain	Day 1	mild to moderate / Recovered
Patient 8	Female	88	26.8	-	Twice	77 Days	317 (AU/mL)	Fever, cough, nausea	Day 0	mild to moderate / Recovered
Patient 9	Female	88	19.9	-	Twice	75 Days	505 (AU/mL)	Fever	Day -1	mild to moderate / Recovered
Patient 10	Female	76	21.4	Diabetes	Twice	54 Days	2884 (AU/mL)	Cough	-	mild to moderate / Recovered
Staff 1	Female	56	20.9	Hypertension, Smoker	Once	180 Days	No data	Fever, cough	Day 2	mild to moderate / Recovered
Staff 2	Female	58	26.7	Diabetes Hypertension	Twice	154 Days	1134 (AU/mL)	Headache, rhinorrhea, loss of taste and smell	Day 7	mild to moderate / Recovered
Family 1 of Staff 2	Male	31	36.2	-	-	-	3.5 (AU/mL)	Fever	Day 3	mild to moderate / Recovered
Family 2 of Staff 2	Male	25	20.8	Bronchial asthma	-	-	2.2 (AU/mL)	Fever, fatigue, diarrhea, Headache	Day 4	mild to moderate / Recovered
Family 3 of Staff 2	Male	22	35.2	Diabetes Hypertension	-	-	0.1 (AU/mL)	Fever	Day 3	mild to moderate / Recovered
Exposure 1	Male	44	23.0	-	-	-	1.3 (AU/mL)	-	-	-
Exposure 2	Female	80	19.0	Diabetes Hypertension	Once	68 Days	625 (AU/mL)	-	-	-
Exposure 3	Female	80	17.4	-	Twice	74 Days	1599 (AU/mL)	-	-	-
Exposure 4	Male	75	20.3	Chronic kidney disease Diabetes Hypertension	Twice	53 Days	No data	-	-	-
Exposure 5	Female	81	15.5	Hypertension, Atrial fibrillation	Twice	77 Days	No data	-	-	-

^a^ As defined by the Centers for Disease Control and Prevention

^b^ The lowest serum IgG titer range from 11 days before to 7 days after onset or diagnosis, SARS-CoV-2 IgG Reagent Kit (Abbott)

^c^ The lowest serum IgG titer range from 5 days to 9 days after Exposure, SARS-CoV-2 IgG Reagent Kit (Abbott)

**Fig 2:**
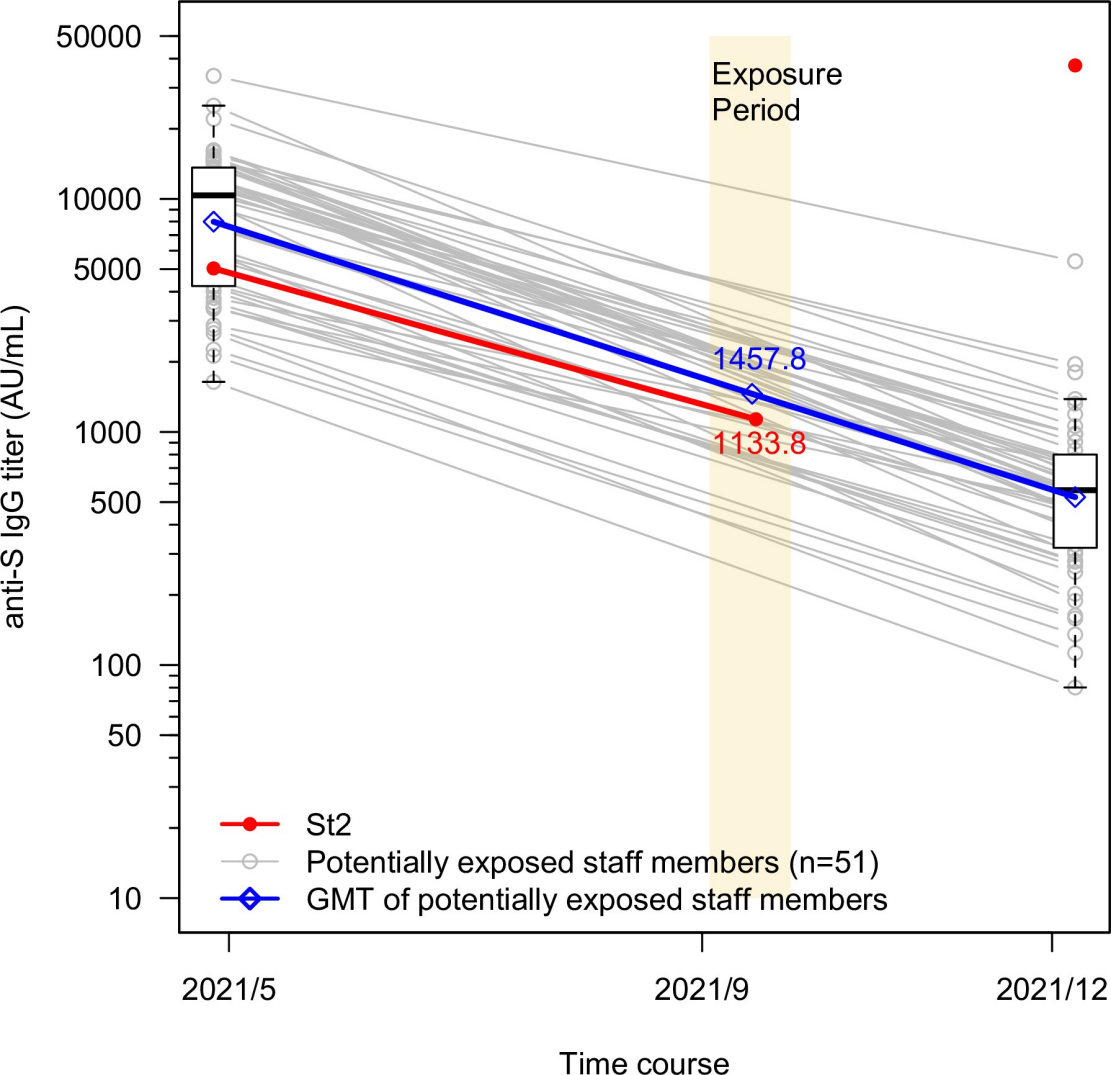
Reduction of anti-S IgG titers after SARS-CoV-2 vaccination in potentially exposed staff members and staff 2. Exposure period, defined as from two days before the onset of the first person to the end of the isolation period of the last person, is highlighted in yellow. In the 51 potentially exposed staff members, the grey circles and lines show anti-S IgG titers and their connection at the measurement points of May and December of 2021 for each. The blue circles and bars indicate the geometric mean titer (GMT) and 95% confidence interval (CI) of the titers at the two points, and the blue line shows the connection of their GMTs. The blue diamond indicates the GMT of estimated anti-S IgG titers calculated on the median day of the exposure period. The red circles and line show the two measured titers and their connection for Staff 2 (St2) with breakthrough infection.

The geometric mean titer (GMT) of the peri-infection anti-S IgG titers in seven of the eight persons with breakthrough infection after two doses was 1,034 AU/ml (95% CI, 398 to 2,686) (Table 1). Of the 486 staff members, including 51 of 105 potential exposures, 289 had two antibody measurement points, before and after the outbreak. None showed any increase in anti-S IgG titers or positivity of anti-N IgG titers between the sampling points (data not shown). Among the 51 potential exposures, the GMT of the measured anti-S IgG titers was 7,986 AU/ml (95% CI, 6,543 to 9,746) at the measurement point after the second dose, decreasing to 525 AU/ml (95% CI, 423 to 653) at the subsequent point at least 6 months later, a decreasing rate of 93.4% (Fig 2). The GMT of the estimated anti-S IgG titers in the exposure period was 1,457.8 AU/ml (95% CI, 1,196 to 1,777), an 81.7% decrease from that of the peak period (Fig 2). Of the 51 potential exposures, 13 (25.5%) had a lower estimated anti-S IgG titer than the measured titer (1,133.8 AU/ml) of the infected staff members (St2) during the exposure period. The results obtained for the 238 unexposed staff members were similar to those of the potential exposures (S1 Fig). A peri-infection anti-S IgG titer was lower than 1,458 AU/ml (an estimated GMT in potential exposures) in five (four inpatients and one staff member) of the seven persons with breakthrough infection. The estimated peak anti-S IgG titers of the four inpatient cases were lower than 7,986 AU/ml (a GMT for the peak period of potential exposures), and all were older than 75 years (S2 Fig).

**Fig 3:**
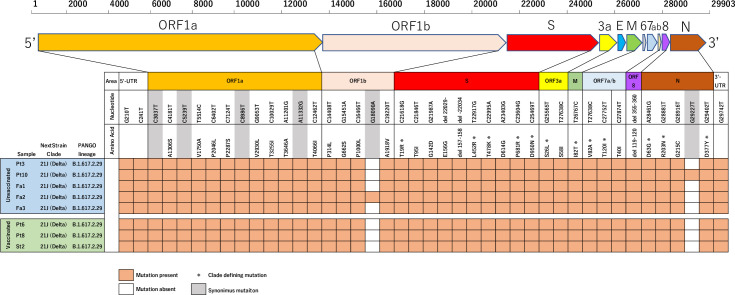
Mutation map of amino acids and nucleotides for the samples from SARS-CoV-2 infected persons. All mutations identified in the whole-genome sequencing are shown. The upper row shows all nucleotide mutations including synonymous mutations. The lower row shows the amino acid mutations caused by nonsynonymous nucleotide mutations.

Nasopharyngeal swab samples were collected from 13 infected persons: nine inpatients, one staff member, and three staff family members. After excepting five samples with high cycle threshold (Ct) values that were considered unreliable, we were able to obtain whole-genome sequence data for eight samples (GISAID Accession ID: EPI_ISL_13321067/13329599/13330152/13330808/13330818/13331001/13331003/13331004). All of the sequences obtained were classified as B.1.617.2.29 (AY.29), a delta (B.1.617.2) variant (Figs 3 and S3). All had the same amino acid mutations as those of the AY.29 variant circulating during the same period in Japan. The amino acid mutations were identical for the vaccinated and unvaccinated persons, with only one nucleotide mutation in two samples from unvaccinated persons.

## Discussion

The associations between SARS-CoV-2-related vaccine effectiveness, humoral immunity, and breakthrough infections are gradually becoming clearer. Goldberg, Y et al. showed waning vaccine effectiveness against delta variant infection after vaccination [1]. Levin, E. G. et al. clarified the antibody kinetics, showing that the anti-S IgG titers linearly declined at a constant rate over at least six months [4]. In other reports, lower anti-S IgG titers tended to be associated with a higher likelihood of SARS-CoV-2 breakthrough infection [8, 9], though no definitive results have been reported on the actual difference in antibody titers between infected and uninfected individuals. In this study, we found that persons with breakthrough infection had a peri-infection anti-S IgG titer lower than the estimated anti-S IgG titer of potential exposures (1,034 vs. 1,458 AU/ml in GMT). Furthermore, most of the infected elderly inpatients showed IgG titers much lower than staff members with potential exposure in both the peri-infection and peak periods. In our previous study, advanced-aged nursing home residents had significantly lower anti-S IgG titers than healthcare workers in the peak period after the second dose [10]. Taken together, our findings indicated that a reduced anti-spike IgG level after SARS-CoV-2 vaccination is an indicator of waning protection against its infection. The methodology used for estimation in our study would be useful for comparing the post-vaccination antibody levels of infected and uninfected persons and for evaluating the risk of SARS-CoV-2 breakthrough infection.

That only 1.9% (2 of 107) of the potential staff exposures became infected seemed relatively low, considering that hospital workers are at a high risk for infection through their close contact with inpatients. Our results showing no anti-S IgG elevation or anti-N IgG seroconversion for potential exposures indicated that we probably did not miss any cases of asymptomatic breakthrough infection. The low infection rate of staff members might be in part attributed to their high vaccination rate, even though their estimated antibody titers may have decreased to a relatively low level at the time of the outbreak compared with their peak value after vaccination. While there may be a threshold value that determines the likelihood of infection, it must be noted that many close and potential exposures who had or were estimated to have a low anti-S IgG titer did not become infected. For example, although the age, vaccination status, and anti-S IgG level were quite similar for the four inpatients in room 2, two became infected and two did not (Table 1 and Fig 1). This outcome indicated that a decreased serum antibody titer is not the only decisive factor in SARS-CoV-2 breakthrough infection. One explanation may be the difference in frequency and duration of contact with an infected person, and another possibility is the influence of cellular immunity related to T cell function. Some reports showed that SARS-CoV-2-specific memory T cell function was relatively maintained, even after six months from the second vaccination [11, 12]. Our preliminary analysis showed that in most potentially exposed staff members, IFN-γ-producing SARS-CoV-2-specific T cells were detected at more than six months after the second vaccination (data not shown).

The sequence results showed little variation in the viral genome among samples obtained during the outbreak, regardless of vaccination status. This probably means that short-term genomic mutations of the genotype circulating in the community that would allow the virus to escape the host immunity elicited by vaccination were not the cause of the breakthrough infection observed in this study. There were no severe cases in the outbreak, which seems fortunate considering the rate of severity from the delta variant. Vaccination and/or the very early introduction of antibody cocktail (Casirivimab/imdevimab) therapy may have contributed to this outcome.

Because we focused on the retrospective real-clinical reports of breakthrough infections of the Delta variant, the number of uninfected and infected individuals with whom antibody titers could be compared was limited and matching for age, sex, and underlying disease was difficult. Because of these factors, no clear conclusions could be drawn in this study regarding the association between SARS-CoV-2 breakthrough infection and antibody titers. Also, neutralizing activity is a direct parameter of humoral immunity, but it was not measured in this study. It has been pointed out that neutralizing activity has different reduction kinetics from antibody titer [4], so further study is needed on this point. Even considering these limitations, we feel that our results would be valuable to physicians dealing with COVID-19 outbreaks. The lack of serum samples from uninfected persons during the outbreak led us to estimate the antibody titer, based on a report that anti-S IgG titer reduction can be plotted as linear in a single-logarithmic graph [4], although this has not been fully validated. Collecting a sufficient number of blood samples at an appropriate timing for measuring antibody levels in uninfected persons is often challenging during the chaos of an outbreak. The use of our methodology for the estimation of antibody levels would be significant for evaluating those levels in uninfected persons during an outbreak. Further research is needed to clarify the dynamics of antibodies after SARS-CoV-2 vaccination and to validate the risk of breakthrough infections.

## Conclusions

We experienced a nosocomial delta variant breakthrough infection in persons who had completed two vaccine doses against SARS-CoV-2. Our findings indicate that the analysis of the measured and estimated specific antibody levels after vaccination would be helpful for evaluating the risk of breakthrough infection. The third (booster) vaccination is now being promoted and the effectiveness has been reported [13]. Measurement and estimation of antibody levels after the initial vaccine series may also be useful for determining the necessity of an additional vaccination.

## Supporting information

S1 Fig Reduction of anti-S IgG titers after SARS-CoV-2 vaccination in unexposed staff members and staff 2. Outbreak period, defined as from two days before the onset of the first person to the end of the isolation period of the last person, is highlighted in yellow. For the 238 staff members, the grey circles and lines show anti-S IgG titers and their connection at the measurement points of May and December 2021 for each. The blue circles and bars indicate the geometric mean titer (GMT) and 95% confidence interval (CI) of the titers at the two points, and the blue line shows the connection of their GMTs. The blue diamond indicates the GMT of estimated anti-S IgG titers calculated on the median day of the exposure period. The red circles and line show the two measured titers and their connection for Staff 2 (St2) with breakthrough infection.
(PDF)

S2 Fig Estimated peak of anti-S IgG titers after SARS-CoV-2 vaccination in inpatients with breakthrough infection. We were able to collect serum samples from the six hospitalized patients with breakthrough infection, at two or more points before their peri-infection period. Each dot indicates the measured anti-S IgG titer. When there were only two sampling points, a line was drawn to connect the points. A line with three or more sampling points was based on the results of linear regression. The triangle shows the timing of onset for each. The predicted peak period, between 21 and 30 days after the second vaccination, is highlighted in yellow.
(PDF)

S3 Fig Location on the phylogenetic tree of the samples from SARS-CoV-2 infected persons. All samples (Blue box) were included in a subclade of the delta (B.1.617.2) variant (Red shading). The phylogenetic tree was obtained from worldwide data on Nextclade in September of 2021.
(PDF)

## Abbreviations

SARS-CoV-2: severe acute respiratory syndrome coronavirus 2
LAMP: Loop-Mediated Isothermal Amplification
NIH: National Institutes of Health
UCSC: University of California Santa Cruz
GMT: Geometric mean titer
anti-S IgG: anti-spike RBD protein IgG
anti-N IgG: anti-nucleocapsid protein IgG
CI: Confidence interval
Ct: Cycle threshold
