# Supplementary figures and images for: Post-vaccination antibody evaluation for nosocomial SARS-CoV-2 delta variant breakthrough infection
Source: PLoS One. 2022 Jul 25;17(7):e0272056. doi: 10.1371/journal.pone.0272056 (PMC9312404; doi:10.1371/journal.pone.0272056)

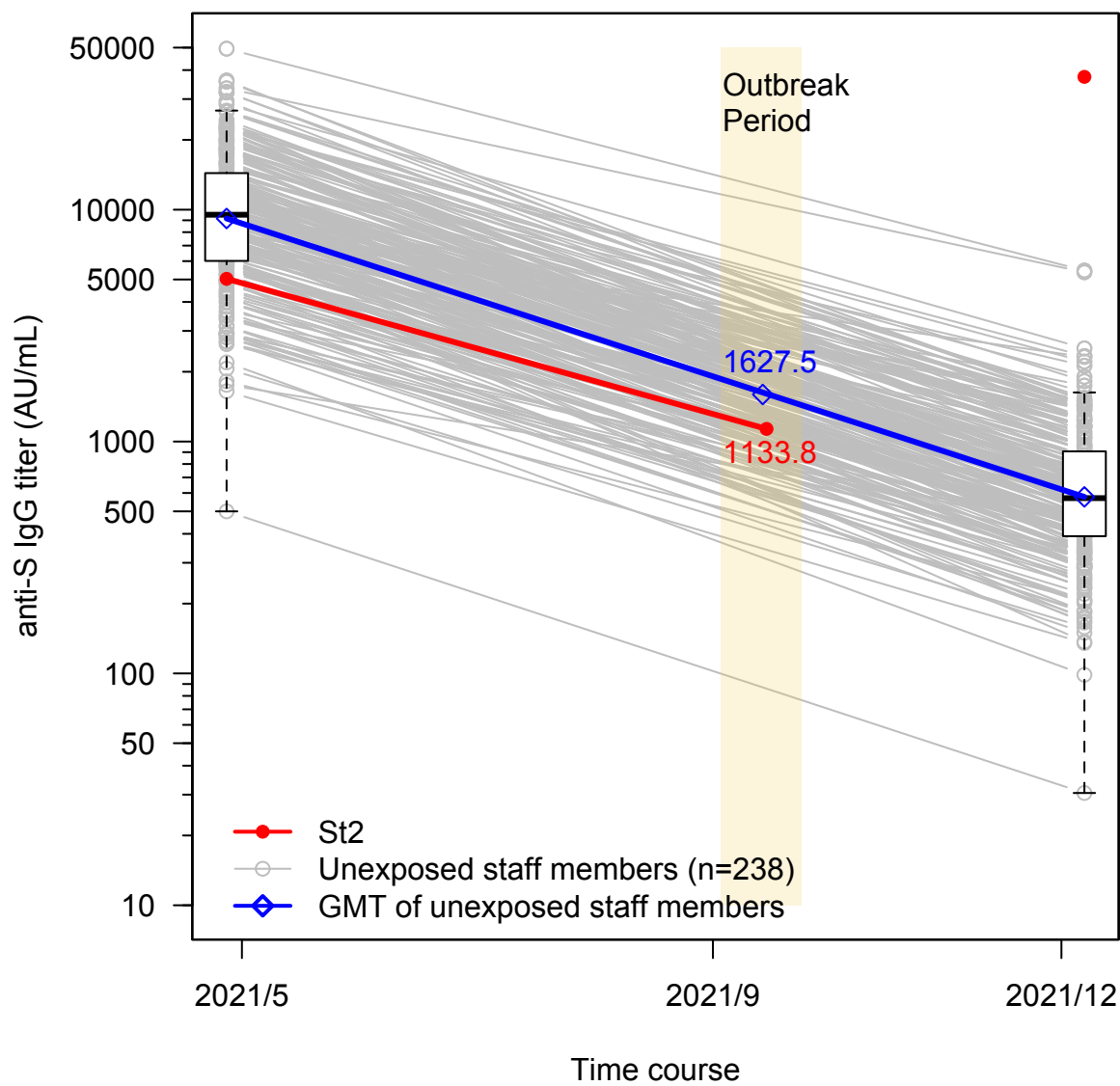

Supplement: S1 Fig — Outbreak period, defined as from two days before the onset of the first person to the end of the isolation period of the last person, is highlighted in yellow. For the 238 staff members, the grey circles and lines show anti-S IgG titers and their connection at the measurement points of May and December 2021 for each. The blue circles and bars indicate the geometric mean titer (GMT) and 95% confidence interval (CI) of the titers at the two points, and the blue line shows the connection of their GMTs. The blue diamond indicates the GMT of estimated anti-S IgG titers calculated on the median day of the exposure period. The red circles and line show the two measured titers and their connection for Staff 2 (St2) with breakthrough infection. (PDF) [file pone.0272056.s001.pdf]

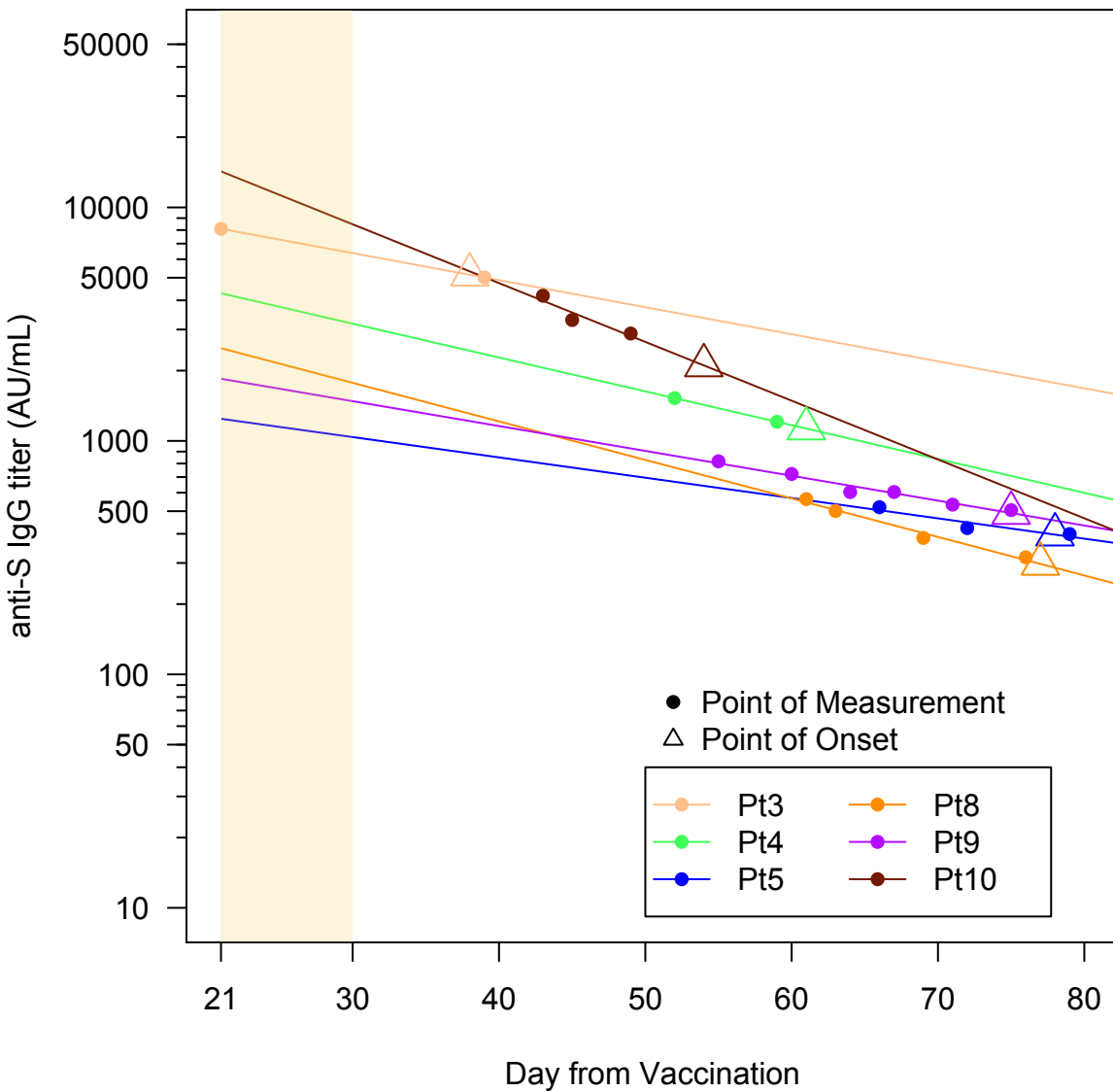

Supplement: S2 Fig — We were able to collect serum samples from the six hospitalized patients with breakthrough infection, at two or more points before their peri-infection period. Each dot indicates the measured anti-S IgG titer. When there were only two sampling points, a line was drawn to connect the points. A line with three or more sampling points was based on the results of linear regression. The triangle shows the timing of onset for each. The predicted peak period, between 21 and 30 days after the second vaccination, is highlighted in yellow. (PDF) [file pone.0272056.s002.pdf]
