# Supplementary material for: Post-vaccination antibody evaluation for nosocomial SARS-CoV-2 delta variant breakthrough infection
Source: PLoS One. 2022 Jul 25;17(7):e0272056. doi: 10.1371/journal.pone.0272056 (PMC9312404; doi:10.1371/journal.pone.0272056)
Supplement: S3 Fig — All samples (Blue box) were included in a subclade of the delta (B.1.617.2) variant (Red shading). The phylogenetic tree was obtained from worldwide data on Nextclade in September of 2021. (PDF) [file pone.0272056.s003.pdf]

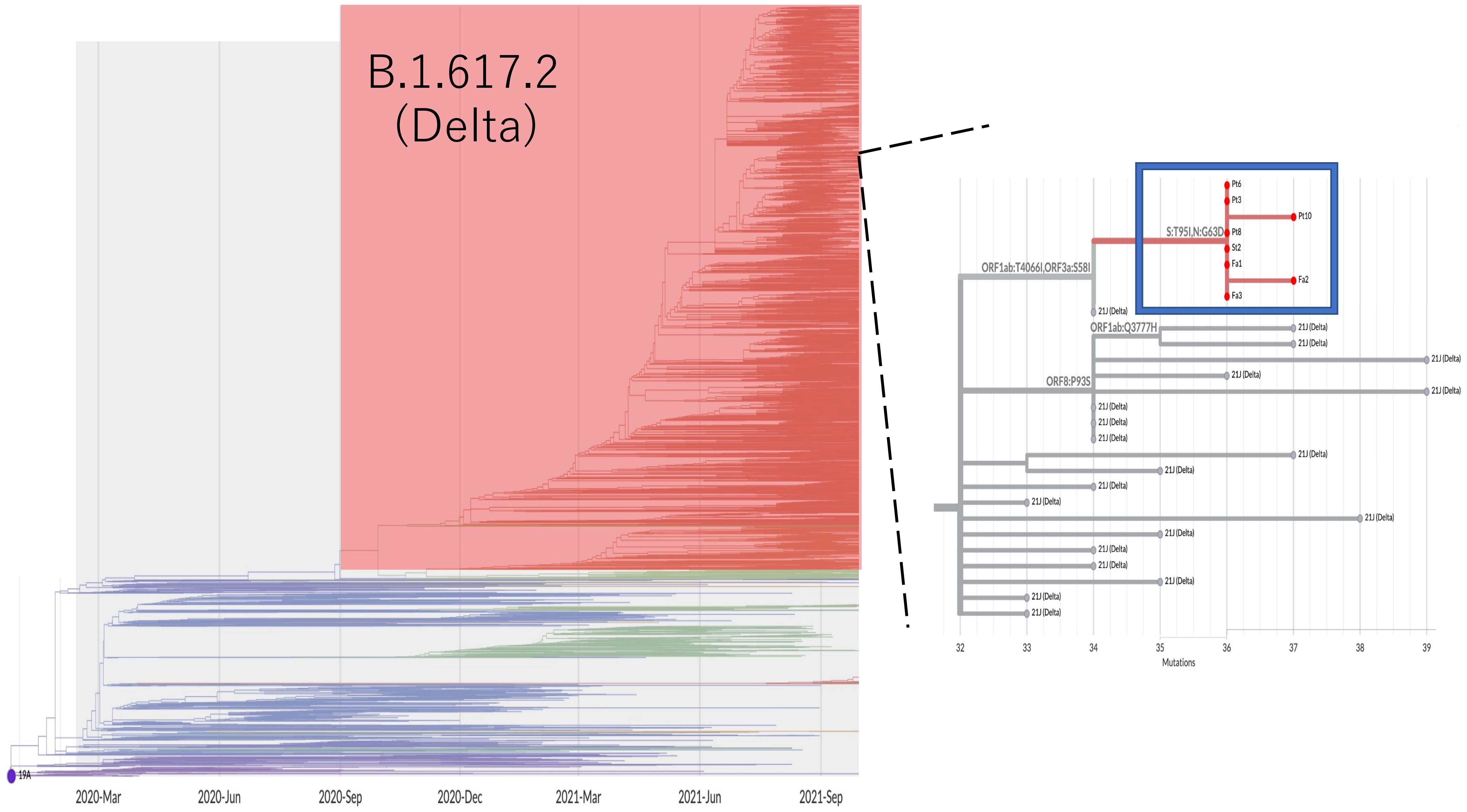

B.1.617.2
(Delta)
19A
2020-Mar
2020-Jun
2020-Sep
2020-Dec
2021-Mar
2021-Jun
2021-Sep
Pt6
Pt3
Pt10
S:T95I,N:G63D
Pt8
St2
Fa1
Fa2
Fa3
ORF1ab:T4066I,ORF3a:S58I
ORF1ab:Q3777H
ORF8:P93S
21J (Delta)
32
33
34
35
36
37
38
39
Mutations
